# Risk Assessment and Management for Living Well with Dementia (1), and Key Issues in Evolving Dementia Care. International Theory-based Policy and Practice (2)

**Published:** 2013-05-07

**Authors:** Gillian Manthorpe

**Affiliations:** NIHR School for Social Care Research, King’s College London, UK E-mail: jill.manthorpe@kcl.ac.uk

Risk assessment and risk management lie at the heart of many professionals’ activities. The production of an accessible good practice guide on Risk Assessment and Management for Living Well with Dementia by Prof. Charlotte Clarke and her colleagues is a welcome reminder that risk taking need not be necessarily feared. Instead it can be one way to improve the quality of life and of death for people with dementia. The strength of this guide is that it rests on authentic experiences and practice encounters in health and social care. This has resulted in an accessible text suitable for people who have attended various ‘risk training’ events and those who have not.

Most professionals, whatever their background, know that decisions about risk in health and social care have to cross service and professional boundaries. They often encounter the anxieties of family carers who may have their own perspectives. These points are sensitively addressed in the first part of the guide. In particular, there are welcome examples of risk-related decisions when a person with dementia has alcohol related problems or the dementia seems to have been related to alcohol misuse.

When many professionals are required to complete risk assessments or monitoring documents on a computer screen, there can be complaints that this becomes rather impersonal. Threaded throughout this book are welcome illustrations that place the person at the centre of the reasoning and decision-making undertaken by professionals. While some risk decision-making is shared between professionals, team work is enhanced by each professional adding something from their own perspective and expertise. This makes assessing and managing risk ideal subjects for integrated working. In many cases the added value of different views is clear.

The second book has a more international dimension with contributions from different parts of the world. The editors have assembled a range of authors to cover the evolution of dementia policy and practice in their own areas. Part One outlines the main conceptual frameworks that lie behind policy and practice understandings of dementia. Rather than being introspective, these are used as a springboard for explaining the action being taken in Canada by pressure groups who wished to increase investment in dementia care and research. The argument is made that the case for such investment had to be more evidence based if it was to be compelling. The task of gathering of evidence about the ‘costs’ of dementia, and where they fall, is work to which many professionals now contribute.

Common to many accounts in this collection is the trend to formulating specific policy documents to address dementia care. Several of the chapters in Part Two of this collection discuss the development of these and the specific approaches taken to compiling a policy initiative and engineering its implementation. These are useful to assemble and different practitioner groups may wish to make further comparisons of the context of the policy initiatives. In England the National Dementia Strategy is well explained by the first Dementia ‘Tsar’, Professor Sube Banerjee (Chapter 5) while Madame Guisset-Martinez and her colleagues (Chapter 6) outline the three French plans on dementia that were constructed 2008–2012. New to many readers will be an account of the development of dementia policy in the small country of Malta by Dr. Charles Scerri in Chapter 8.

Part Three of this collection moves to discuss innovative practice. This includes discussions of memory clinics in Scotland, development of public health approaches and services in India, environmental design in residential care in Australia, and training for nurse aides in the deep South of the US that acknowledges racial, economic and gender dimensions of staff and service users. These rather eclectic innovations place some flesh on the policy bones established in Part Two of the book. An integrated approach is specifically noted as important in linking theory and practice in dementia care and this stance underpins the collection as a whole.

Both these books are aimed at professionals and researchers from a variety of disciplines and agencies. In thinking about integrated care we might consider the role of textbooks that are read by people from different academic traditions. One way in which dementia care has developed does seem to be learning materials that are used across different professional groups. This would seem to foster inter-disciplinary understanding and the interesting material contained in both these books would seem to suggest that this area of practice is likely to be receptive to further specific steps along the path of integration.

